# Health care needs, eHealth literacy, use of mobile phone functionalities, and intention to use it for self-management purposes by informal caregivers of children with burns: a survey study

**DOI:** 10.1186/s12911-023-02334-w

**Published:** 2023-10-23

**Authors:** Fatemeh Rangraz Jeddi, Ehsan Nabovati, Mohammadreza Mobayen, Hossein Akbari, Alireza Feizkhah, Joseph Osuji, Parissa Bagheri Toolaroud

**Affiliations:** 1 Health Information Management Research Center, Kashan University of Medical Sciences, Kashan, Iran; 2 Department of Health Information Management & Technology, Kashan University of Medical Sciences, Kashan, Iran; 3 Burn and Regenerative Medicine Research Center, Guilan University of Medical Sciences, Rasht, Iran; 4 Social Determinants of Health (SDH) Research Center, Department of Biostatistics and Epidemiology, School of Public Health, Kashan University of Medical Sciences, Kashan, Iran; 5 Department of Medical Physics, School of Medicine, Guilan University of Medical Sciences, Rasht, Iran; 6 School of Nursing and Midwifery, Faculty of Health, Community, and Education, Mount Royal University, Calgary, Ab Canada

**Keywords:** Burns, Informal caregivers, Literacy, Needs Assessment, Mobile Health

## Abstract

**Background:**

This study aimed to assess health care needs, electronic health literacy, mobile phone usage, and intention to use it for self-management purposes by informal caregivers of children with burn injuries.

**Methods:**

This cross-sectional research was carried out in 2021 with 112 informal caregivers of children with burns in a burn center in the north of Iran. The data collection tools were questionnaires that included the participants’ demographics, their E-Health Literacy, their current mobile phone usage, and their desires for mobile phone use for burn care services.

**Results:**

Most informal caregivers had smartphones (83.0%) and Internet access (81.3%). Most participants occasionally used phone calls (63.4%), the Internet (45.5%), and social media (42.9) to receive information about psychosocial disorders, infection control, wound care, pain, itch, physical exercise, and feeding. Most participants have never used some of the mobile phone functionalities to receive burn-related information, such as applications/Software (99.1%) and e-mail (99.1%). Nevertheless, most informal caregivers desire to use mobile applications for self-management purposes in the future (88.4%). The mean eHealth literacy score was 25.01 (SD = 9.61). Informal caregivers who had higher education levels, access to the Internet, and lived in urban areas had higher eHealth literacy (P < 001).

**Conclusion:**

The current research delivers beneficial information about the healthcare needs of informal caregivers and their preference to use mobile functionality to receive burns-related healthcare and rehabilitation information post-discharge. This information can help design and implement mobile health (mHealth) interventions to enhance the self-care skills of informal caregivers.

**Supplementary Information:**

The online version contains supplementary material available at 10.1186/s12911-023-02334-w.

## Introduction

Burn injuries in children are distressing physical and emotional events with long-term outcomes [1]. More than half a million children worldwide are hospitalized with burn injuries in hospitals annually, most of which occur in developing countries [2]. A systematic review (2017) revealed that the prevalence of burns in Iran in children under 15 years of age varies from 5.9 to 50 cases per 100,000 children [3]. Recently, advances in medical science have led to improved clinical outcomes and reduced mortality in children with burns. However, the mortality rates in these patients are still high [4–6]. Previous evidence has shown that children with burns and their families experience physical and socio-psychological problems after discharge [7–9]. The lack of knowledge of patients and informal caregivers about burn self-care exposes them to risks such as neuropathy, contracture, deformity, and readmission of patients to medical centers [10, 11].

One of the methods to improve the self-management abilities of patients with burns and their caregivers (i.e., providing demographic and clinical information about wound care, scar management, mental health, nutrition, rehabilitation, pain, and itch management) is to use information technology tools (e.g., the Internet, mobile phones, and computer software) [12]. With recent advances in technology and increasing access to smartphones and mobile health (mHealth) tools among low-income people, it is possible to provide self-care education more effectively by providing support services such as communications, education, and reminders to encourage healthy behavior [13–15]. mHealth, as a component of electronic health, is related to the use of mobile phones and other wireless technologies to improve the provision of health-related services. Previous studies have shown that mobile health tools, such as text messaging, applications, and other relevant technology, can help patients communicate better and receive better health care [16, 17]. Moreover, the potential advantages of mobile health technologies for prevention, diagnosis, and therapy include usability, mobility, availability in all regions, increased access to health care, and lower costs. [18].

Although there is insufficient research on the effects of mHealth tools on burn outcomes, two studies have shown that these interventions positively empower parents in caregiving for their children, improve their quality of life (QOL), and positively reduce unplanned hospital readmissions [19, 20]. One of the critical factors for successfully implementing mobile health interventions and using mobile health applications effectively is considering patients’ e-health literacy skills and attitudes toward these technologies [14, 21, 22]. Given that these variables may be influenced by culture, and considering the cultural and ethnic diversity in Iran, we are investigating this study on a specific culture. This study aimed to investigate health care needs, eHealth Literacy, mobile phone usage, and the intention to use it for self-management in informal caregivers of children with burn injuries.

## Materials and methods

### Study design, setting, and population

The present cross-sectional study was conducted in 2021 in a burn center in the north of Iran. This center is the only burn center in Guilan province, with 55 beds in the burn department and ten beds in the ICU ward, and it has approximately 700 admissions per year, covering all burn patients in the region. This study surveyed health care needs, eHealth literacy, mobile phone usage, and intention to use for self-management purposes by informal caregivers of children with burns. In a similar study by Liang et al. [8], the mean and standard deviation (SD) of burn patients’ total healthcare needs score after discharge was 71.4 ± 24. The minimum required sample size of 110 participants was computed using the 95% confidence interval and a 4.5 accuracy in calculating the mean score.

### Data collection

The research was conducted with the informal caregivers of discharged burn children under 18 who came for follow-up or wound dressing to the burn and plastic surgery clinic in the first month after discharge, between April and November 2021. Two researchers visited the burn and plastic surgery clinic daily during the study period to collect the data. Parents of children with burns who qualified were asked to participate in the study. If informal caregivers volunteered to participate in the study, they were given brief face-to-face explanations about the study objectives and how to complete the questionnaire in the waiting room. The paper questionnaires were delivered with an informed consent letter to the participants. Participants were informed that they could withdraw from the study at any moment and that their answers would remain private. The participants completed the questionnaires in the researcher’s presence so that the researchers could resolve any ambiguities. Participants identifying information was removed from the questionnaires to ensure confidentiality. Also, each questionnaire was given an identification code to collect clinical information (percentage and cause of burns) and match it with the completed questionnaires (ID code). The identification code of the informal caregivers was the medical record number of their children so that the researcher could access the patient’s clinical information using the hospital information system (HIS). A total of 153 informal caregivers of children with burns attending the burn and plastic surgery clinic were identified, of whom 114 were volunteers to complete the scale, with a response rate of 74.51%. The information related to two patients was deleted after the study was finished because of incompleteness and lack of access to the patients at the end of the study. A total of 112 participants were included in the study.

### Questionnaires

#### Demographic and clinical information

Information collected included sex, age, marital status, education level, place of residence, socio-economic level, total body surface area (TBSA), and the primary cause of burns.

#### Informal caregivers’ healthcare needs

The researchers developed the questionnaire based on the literature review to determine the healthcare needs of informal caregivers post-discharge [8, 23, 24]. In order to assess face validity, first, the questionnaire was given to 15 caregivers of children with burns, and they were asked to comment on the level of difficulty, relevancy, and ambiguity of questions. The participants’ opinions about the items were carefully recorded, and they were asked to explain more where necessary. Finally, with the advice of the two experts (a burn surgery fellowship and a clinical research nurse), changes were applied to the questionnaire, and face validity was approved. The content validity of the questionnaire was assessed by a 15-member panel consisting of faculty members of health information management, burn specialists, and clinical instructors in the burn unit. The validity of each item (i.e., relevance, clarity, and simplicity) plus the validity index of the entire tool was determined based on a four-point Likert scale from “unfavorable” (score of 1) to “totally favorable” (score of 4). The content validity of each item was assessed based on the content validity index (CVI), and if less than 0.7, the item was eliminated. The content validity ratio (CVR) was also determined based on Lawshe’s Table [25], and the items with values less than 0.49 were eliminated. The reliability of the questionnaire was determined by Cronbach’s alpha, which was 0.81. Among all the items, 6 items were deleted, and the final approved checklist related to caregiver needs assessment comprised ten sections (Additional file 1), including (1) taking medication (7 items), (2) control of infection and wound care (7 items), (3) bathing (3 items), (4) getting dressed (2 items), (5) physical exercise is (4 items), (6) nutrition (5 items), (7) itch (1 item), (8) pain (2 items), (9) psychosocial needs (8 items), and (10) follow up (2 items). Participants responded to the items of this tool based on a five-point Likert scale from “not at all” (score of 1) to “very much” (score of 5). Higher scores indicate more information needed for health care.

#### **Use of mobile phone functionalities and desire to use**

The questionnaire was prepared based on experts’ opinions about information relevant to burns and mobile phone usage. Two specialists in health information management and medical informatics reviewed and confirmed the face validity of the questionnaire. The content validity of the questionnaire was checked by a 12-member panel consisting of Faculty members of health information management, medical informatics, and burn specialists. Each item’s content validity was evaluated using the Content Validity Index (CVI), and if the CVI was less than 0.7, the item was revised. Also, the Content Validity Ratio (CVR) was determined based on Lawshe’s Table, and the items with values less than 0.56 were eliminated. The scale reliability was determined by Cronbach’s alpha which was 0.83. The final questionnaire, which contained 39 items (Additional file 2), was divided into two main sections: (1) overall use of mobile phone functionalities (24 items) and (2) willingness to use mobile phone features (15 items). Five items had a “Yes” or “No” response, and six items had four options (every day, several times per week, occasionally, and never). Also, 20 items had seven options (phone/voice call, SMS, Email, internet search, social media, software/applications, and none), and eight had six options (phone/voice call, SMS, Email, social media, software/applications, and none).

#### E-Health literacy scale

This study used the eight-item eHealth literacy scale (eHEALS) to measure perceived eHealth literacy (Additional file 3). It is an 8-item scale designed to assess individuals’ knowledge, comfort, and perceived skills at using digital health technology to find, assess, and utilize electronic health information to solve a health problem (internal consistency reliability = 0.88 and test-retest reliability = 0.40 to 0.68) [26]. The eHealth literacy scale is scored on a 5-point Likert scale with response options ranging from “strongly disagree” (score = 1) to “strongly agree (score = 5)”. The score range of this questionnaire was from 8 to 40. This tool has been applied internationally as a patient-reported outcome measure for digital health interventions [27]. Bazm et al. has examined the content and face validity of the Iranian translation of the eHealth literacy questionnaire. Also, they confirmed the reliability of this tool using Cronbach’s alpha coefficient of 0.88 [28]. The present study used an Iranian-translated version of Bazm et al.‘s questionnaire.

### Statistical analysis

A total of 114 informal caregivers participated in this study. However, at the study’s end, two participants were excluded because of incomplete information and a lack of access to them. A total of 112 participants were included in the study, and there was no missing data for these participants. The Statistical analysis was performed using the SPSS software package (version 16.0, SPSS Inc., Chicago, IL, USA). Quantitative and qualitative variables were presented using mean (standard deviation) and number (percentage). The normality of the data was assessed using the Kolmogorov-Smirnov test. Due to the normal distribution of data, analysis of Variance and independent t-test were used to assess associations between eHealth literacy scores and sociodemographic characteristics. Also, Post hoc analysis using the Scheffé post hoc criterion was used to explore differences between specific groups. The significance level was considered less than 0.05.

## Results

### Participants

A total of 112 participants were included in the study. Of the informal caregivers, 93 (83.0%) were female. The mean age of participants was 34.79 (SD = 8.15) years. A total of 67 informal caregivers (59.8%) had a Diploma’s degree or lower, and 71 (63.4%) lived in the city. The average age of the children was 6.35 (SD = 5.11) years. The most frequent burn etiology was Hot liquid, steam, or gas, which occurred in 52.7% of the children. The mean TBSA was 23.96 (SD = 8.27).

### Informal caregivers ' healthcare needs

Table 1 shows the healthcare needs of participants. Most participants claimed that their issues are caused mainly by a lack of information regarding physical activity (4.12 [SD = 0.82]) and infection control with wound care (4.07 [SD = 0.75]).

**Table 1:** Healthcare needs in informal caregivers of children with burns after discharge

Items	Healthcare needs of informal caregivers
Medication use	3.22 ± 0.88 (1–5)
Control of infection and wound care	4.07 ± 0.75 (1–5)
Bathing	3.66 ± 0.97 (1–5)
Clothing	3.40 ± 1.02 (1–5)
Physical exercise	4.12 ± 0.82 (1–5)
Nutrition	3.69 ± 1.17 (1–5)
Itch	3.91 ± 0.99 (1–5)
Pain	3.76 ± 0.96 (1–5)
Psychosocial needs	3.79 ± 0.89 (1–5)
Follow up	3.20 ± 0.99 (1–5)

Data are presented as mean (standard deviation).

### Overall use and intention to use mobile phone functionalities

Table 2 shows the overall use and desire to use mobile phone functionalities. Out of the 112 participants, 93 (83.0%) had smartphones, 79 (70.5%) used mobile phones to receive burn care services, 91 (81.3%) had access to the Internet, and 1 (0.9%) had one burn-related application on their smartphone. Also, 99 informal caregivers (88.4%) desired to use mobile phones to get educational information.

**Table 2:** Overall use and intention to use of mobile phone functionalities by informal caregivers of children with burns

Items	Number (%)	
	Yes	No
Use of mobile phones to receive burn care services	79 (70.5)	33 (29.5)
Mobile internet access	91 (81.3)	21 (18.8)
Have a smartphone (ability to install applications)	93 (83.0)	19 (17.0)
Installing mobile applications related to burn care	1 (0.9)	111 (99.1)
Desire to use mobile phones to receive burn care services	99 (88.4)	13 (11.6)

Data are presented as numbers (percentages).

### The frequency of use of mobile phone functionalities

Table 3 shows the frequency of use of mobile phone functionalities by informal caregivers of children with burns to receive burn-related information in four categories: every day, several times per week, occasionally, and never. The results showed that most participants stated that they have never used some of the mobile phone functionalities to receive burn-related information, such as applications/Software (99.1%), Email (99.1%), and SMS (79.5%). However, they occasionally used phone calls (63.4%), the Internet (45.5%), and social media (42.9%) to search for and access burn-related information.

**Table 3:** The frequency of use of mobile phone functionalities by informal caregivers of children with burns (N = 112)

Items	Number			
	Everyday	Several times per week	Occasionally	Never
Receiving burn-related information through mobile phone calls (friends, relatives, doctors, and nurses)	0 (0)	15 (13.4)	71 (63.4)	26 (23.2)
Receiving burn-related information through SMS (friends, relatives, doctors, and nurses)	0 (0)	5 (4.5)	18 (16.1)	89 (79.5)
Using mobile Internet to search for burn-related information	3 (2.7)	15 (13.4)	51 (45.5)	43 (38.4)
Using social media (such as Telegram channels, WhatsApp channel, Instagram and …) to access burn-related information	1 (0.9)	6 (5.4)	48 (42.9)	57 (50.9)
Using Email to communicate with others (friends, relatives, doctors, and nurses) to receive burn-related information	0 (0)	0 (0)	1 (0.9)	111 (99.1)
Using of applications to access burn-related information	0 (0)	0 (0)	1 (0.9)	111 (99.1)

Data are presented as number (percentage).

### Use and desire to use mobile phone functionalities for receiving information as reminders and warnings in caregivers of children with burns

Informal caregivers had the most effective use of the Internet search to receive information about psychosocial disorders (53.6%), control of infection and wound care (52.7%), pain (50.9%), itch (44.6%), physical exercise (43.8%), and feeding (40.2%). Also, they used social media most to control infection and wound care (41.1%). The participants had made their greatest use of phone/voice call for psychosocial disorders (47.3%), pain (43.8%), and itch (42.0%). Meanwhile, the informal caregivers had a greater desire to use internet search, mobile applications, and social media (compared to the other functionalities of mobile phones) to receive information about infection control and wound care (Additional file 4).

### Relationship between E-health literacy and demographic characteristics

Based on Table 4, informal caregivers of children with burns showed moderate levels of eHealth literacy, with a mean eHealth literacy score of 25.01 out of 40 (SD = 9.61). The results of the one-way analysis of Variance (ANOVA) showed that eHealth literacy was associated with age intervals (P = 0.041). Post hoc analyses using the Scheffé post hoc criterion indicated that the average eHealth literacy score was lower in the age interval ≥ 40 years (M = 21.57, SD = 9.42) than in other age groups. Regarding the education level, the results show a significant difference between e-health literacy scores and education levels (P < 001). According to the post hoc analysis, participants with lower education levels had a lower E-health literacy score than others. There was a significant difference between eHealth literacy and place of residence (P < 001). Informal caregivers of children living in urban areas had higher scores than those living in rural areas. Also, there was a significant difference between eHealth literacy with a smartphone and mobile internet access (P < 001). There was no significant difference between mean eHEALS scores with sex, marital status, economic status, TBSA, and primary cause (P > 0.05).

**Table 4:** Comparison of E-health literacy score based on basic characteristics

		Health literacy	
	N (%)	E-health literacy score	P value
**Sex**			
Male	19 (17.0)	22.31 (SD = 9.95)	0.180
Female	93 (83.0)	25.56 (SD = 9.50)	
**Age (years)**			
<29	27 (24.1)	24.30 (SD = 9.92)	P < 041
30–39	57 (50.9)	27.05 (SD = 9.18)	
≥40	28 (25.0)	21.57 (SD = 9.42)	
**Marital status**			
Married	110 (98.2)	25.11 (SD = 9.54)	0.415
Other	2 (1.8)	19.50 (SD = 15.56)	
**Education level**			P < 001
High school or less	20 (17.9)	14.05 (SD = 7.81)	
Diploma	47 (42.0)	22.00 (SD = 7.51)	
Associate’s and Bachelor’s degree	37 (33.0)	32.10 (SD = 3.77)	
Master of Science or higher	8 (7.1)	37.37 (SD = 3.29)	
**Place of residence**			
City	71 (63.4)	28.08 (SD = 8.30)	P < 001
Village	41 (36.6)	19.70 (SD = 9.50)	
**Self reported economic status**			0.277
Low	12 (10.7)	21.08 (SD = 10.52)	
Moderate	99 (88.4)	25.43 (SD = 9.47)	
Excellent	1 (0.9)	31.00 (-)	
**Burn percent (TBSA)**			
>20	36 (32.1)	24.30 (SD = 11.73)	0.913
20–29	58 (51.8)	25.05 (SD = 8.26)	
30–39	13 (11.6)	26.30 (SD = 7.89)	
40>	5 (4.5)	26.40 (SD = 13.68)	
**Cause of burn**			
Flame	33 (29.5)	24.36 (SD = 9.86)	0.828
Hot surface	18 (16.1)	22.77 (SD = 10.69)	
Chemical	59 (52.7)	26.00 (SD = 7.07)	
Hot liquid, steam or gas	2 (1.8)	26.03 (SD = 9.26)	
**Have a smartphone**			
Yes	93 (83.0)	26.65 (8.92)	P < 001
No	19 (17.0)	17.00 (9.00)	
**Mobile internet access**			
Yes	91 (81.3)	27.05 (8.66)	P < 001
No	21 (18.8)	16.19 (8.63)	

Among the eight questions related to the eHealth literacy scale, the statements “identifying high-quality health resources from low-quality health resources” (54.5%) and “evaluation skills of health resources on the Internet” (52.7%) had the highest level of disagreement (Additional file 5).

## Discussion

This is the first study investigating health care needs, eHealth literacy, use, and desire to use mobile phone functionalities in informal caregivers of children with burn injuries. In the present study, most informal caregivers of children with burns stated that after discharge, they face problems regarding how to perform physical activity, infection control, and wound care. The majority of the informal caregivers had smartphones and access to the Internet. Most participants occasionally used phone calls, social media, and the Internet to receive burn-related information. Participants used the Internet and social media the most to receive information about psychosocial disorders, infection control, wound care, pain, itch, physical exercise, and feeding. Most participants have never used some of the mobile phone functionalities to receive burn-related information, such as applications/Software, Email, and SMS. However, most informal caregivers desire to use mobile applications to receive reminders regarding medication use and warnings about the risks of not doing any burn-related rehabilitation programs.

The present study showed that most informal caregivers of children with burn injuries require information and training in some areas, such as medication use, wound care, bathing, wearing clothes, physical exercise, feeding, itch, pain, and psychosocial concerns. However, participants’ most common health care needs included physical exercise, infection control, and wound care. A systematic review in 2019 found that informal caregivers need information and support through all stages of treatment and during periods of emotional distress [29]. In a qualitative study carried out in Sweden, the researchers revealed that parents of children with burns had problems with dressing and cleaning wounds, returning to their old lives, returning to school, and having body-image issues [30]. Therefore, It is essential to educate burn self-care about the issues that patients and their informal caregivers will face following discharge from the hospital. Despite providing self-care training during discharge to patients and their informal caregivers, the findings of this study indicate significant informal caregivers ’ healthcare needs post-discharge. This may happen because patients’ educational instructions post-discharge may be lost, not understood completely, or not considered desirable.

Even among underserved communities, the use of digital technologies has grown gradually. The present study shows that many informal caregivers had access to smartphones and the Internet on their mobile phones, consistent with studies showing increasing access to digital technologies [17, 31, 32]. In contrast, a study in Nigeria [33] showed that just 33.5% of patients had mobile internet usage. This finding may be explained by the study participants’ limited buying power and high data charges.

The current study shows that most participants do not use E-mail communication and SMS to receive burn-related information. In contrast, a study in Michigan found that most patients used SMS and E-mails at least once a week [34]. This discrepancy may be due to the lack of knowledge in the current study population about the features of this technology in communicating, not having an email account, late response, and their low literacy level.

The most common topics for which informal caregivers of children needed mobile services included learning how to do physical exercises, infection control, wound care, and warnings about non-completion rehabilitation programs. Other common areas include; medication reminders, pain control, itching control, psychological disorder, dressings, taking baths, and feeding advice. In the present study, more than two-thirds of informal caregivers occasionally used mobile phone calls and the Internet to receive burn-related information. A study in Portugal shows that about half of people search for health information on the Internet [35]. In Spain, most patients use the Internet to receive medical information about their illnesses before seeing a doctor [36].

The present findings showed that informal caregivers used mobile phone capabilities through an internet search, followed by social media, to get burn-related information about their children, which is consistent with the results of other studies [17, 37]. Therefore, it is recommended that medical professionals be familiar with websites and channels with valid information in burn care or design and develop educational applications, online channels on social networks, and websites containing helpful information.

Studies have shown that self-care applications developed for burn patients have promoted healthy behaviors, facilitated self-care management, and improved health outcomes [38–40]. However, most participants in this study did not have a burn-related application installed on their mobile phones. This could be due to the country’s lack of natively designed burn software, poor finances, privacy concerns, and lack of awareness of the programs available in the applications stores, which can be barriers to downloading or using medical applications. Nevertheless, most of the informal caregivers in the present study stated their intention/ willingness to use burn applications for self-management purposes. These results were consistent with findings from previous studies conducted in Ethiopia [31]. A study in New York showed that most adolescents or their informal caregivers desired to use mobile apps, but just a few participants had health-related applications on their cell phones. [41].

A crucial part of health literacy is eHealth literacy. Identifying and evaluating patients’ eHealth literacy levels is the first step in developing strategies to improve patient health knowledge and empower them for positive actions. This is the first study to evaluate the eHealth literacy of informal caregivers of children with burns. Therefore, comparing our findings with other studies is difficult. In this study, the average eHealth literacy score of informal caregivers of children with burns was 25.01 (SD = 9.61). Similarly, a study in Australia reported that the average eHealth score of parents of children with complex CHD was 27.46 (5.47) [42]. Moreover, a study in Iran showed that the mean eHealth literacy score of family informal caregivers of medically ill elderly was 26.16 (SD = 8.83) [43]. Nonetheless, the eHealth literacy score obtained in this study was slightly lower than the studies conducted in some countries [44, 45], which may be because of the difference in healthcare needs, education levels, lack of centralized internet sources, and lack of confidence in it.

In this study, no significant differences were observed between sex and the eHealth literacy score, which was in line with previous studies [46–48]. The findings showed a significant difference between age and e-Health literacy, which is well-known and in line with other authors [43, 48]. In the present study, the correlation results showed that with increasing education levels, e-Health literacy also increases. This finding is similar to other studies in different populations [43, 49]. The mean eHealth literacy scores were higher among participants accessing the Internet than others. This is consistent with a study in the United States [45]. Also, informal caregivers reported important challenges, including evaluating the health resources and problems identifying high-quality health resources from low-quality, similar to studies in Australia [42] and in Florida [49]. These findings can be explained by arguing that people with greater levels of education have better critical and deductive reasoning abilities than others, leading to better judgment and greater confidence in mobile health technologies. Therefore, it is suggested that practical and user-friendly applications for health promotion be designed according to the e-health literacy level of patients. In addition, previous studies conducted in various contexts have shown that targeted educational interventions can increase eHealth literacy and people’s confidence in using these technologies, regardless of age, race, level of education, and prior internet usage [42, 50].

### Strength and limitations

Based on the literature review, the present study is the first study conducted in a developing country about mobile phone usage and the desire to use it by informal caregivers of children with burns to receive self-care services. These findings could provide a basis for future research on designing and developing mHealth interventions for burn patients. The current study had a sample of 112 informal caregivers of children with burns in a single center, which could be considered a limitation. Additionally, all the informal caregivers were chosen from one center because it was the only burn center in Guilan province. The findings cannot be generalized to all informal caregivers of children with burns after discharge because of the sample size and recruitment method. A limitation of this study is the presentation of associations in a univariate manner, acknowledging the inherent lack of independence among certain variables. Given the small sample size, we refrained from employing regression models to avoid potential instability. Therefore, our results should be interpreted with caution. Hence, for better generalizability, future research with larger samples and multivariate analyses is needed to delve deeper into these associations and identify independent predictors of eHealth and general health literacy.

### Implications for practice and future research

The current study specified the healthcare needs of children’s informal caregivers and their preferred types of mobile phone capabilities for acquiring burn self-care services. Designing prospective interventions based on these requirements seems crucial. However, the research findings can still guide clinical specialists to meet the healthcare needs of discharged burn patients and their informal caregivers using mhealth tools. Based on the data, most informal caregivers in this study did not use applications because of a lack of understanding and confidence about the information content of these tools. Therefore, the following activities are suggested: clinical specialists should assure informal caregivers about the quality of the information provided through mobile phones. In addition, specialists should encourage them to use these tools. Furthermore, practitioners should educate caregivers about the potential risks of cell phones, such as the transmission of pathogenic microorganisms.

## Conclusion

The present study showed that many informal caregivers of children with burns use the Internet, mainly through smartphones, to research health care information. Since informal caregivers of children with burns do not have access to their medical professionals after discharge, they need the proper education to learn how to manage their difficulties and find new ways to live with a chronic condition. Hence, it is essential to meet the educational needs of families by using alternative and efficient approaches. mHealth tools are critical to patient self-care and can potentially meet these purposes. To build helpful m-health tools for patients or their informal caregivers, physicians and nurses must focus on the needs and preferences of patients. Also, to encourage more m-health adoption, patients should be aware of the benefits of m-health tools and be actively involved in developing these technologies. Practical and user-friendly health promotion applications should be developed according to the e-health literacy level of patients.

### Electronic supplementary material

Below is the link to the electronic supplementary material.

Additional file 1: Health Care Needs Questionnaire.

Additional file 2: Use of mobile phone functionalities and intention to use for self-management purposes by caregivers of children with burns.

Additional file 3: eHealth Literacy Questionnaire.

Additional file 4: Use and desire to use mobile phone functionalities for receiving information, as reminders and warnings in caregivers of children with burns (N=112).

Additional file 5: Distribution and frequency of eHealth Literacy Scale (eHEALS) scores.

## Data Availability

The datasets used and/or analyzed during the current study are available from the corresponding author upon reasonable request.

## Abbreviations

mHealth: Mobile health
QOL: Quality of life
SD: Standard deviation
eHEALS: eHealth literacy scale
CVI: Content Validity Index
CVR: Content validity ratio
HIS: Hospital information system
