# Supplementary material for: Health care needs, eHealth literacy, use of mobile phone functionalities, and intention to use it for self-management purposes by informal caregivers of children with burns: a survey study
Source: BMC Med Inform Decis Mak. 2023 Oct 23;23:236. doi: 10.1186/s12911-023-02334-w (PMC10591411; doi:10.1186/s12911-023-02334-w)
Supplement: Supplementary file 1 — Additional file 1: Health Care Needs Questionnaire. [file 12911_2023_2334_MOESM1_ESM.docx]

**Additional file 1: Health Care Needs Questionnaire**

| **Items** | | **Reply** | | | | |
| --- | --- | --- | --- | --- | --- | --- |
| **Medication use** | | Not at all | Little | To some extent | Rather much | Very much |
| 1 | How much information is required about the importance and necessity of taking prescribed medications (for example, ointments, pills) in healing a child's burn wound? |  |  |  |  |  |
| 2 | How much information is required about the consequences of not taking the drug on time? |  |  |  |  |  |
| 3 | How much information is required about the medications prescribed? (How much, how often, and how long should the medicine be taken). |  |  |  |  |  |
| 4 | How much information is required about the probable side effects of prescription drugs? |  |  |  |  |  |
| 5 | How much information is required about reducing the side effects of prescription drugs? |  |  |  |  |  |
| 6 | How much training is required if the child refuses to take medication? |  |  |  |  |  |
| 7 | How much reminder is required to give the child's medication on time? |  |  |  |  |  |
| **Dressings, control of infection, and wound care** | | Not at all | Little | To some extent | Rather much | Very much |
| 1 | How much training is required to get on how to dress (change) the grafted area? |  |  |  |  |  |
| 2 | How much information is required about the dressing types and the price-effectiveness ratio? |  |  |  |  |  |
| 3 | How much information is required about the prevention, symptoms, and control of burn wound infection? |  |  |  |  |  |
| 4 | How much information is required about ways to prevent bedsores in children? |  |  |  |  |  |
| 5 | How much information is required about preventing scars and Contracture? |  |  |  |  |  |
| **6** | How much information is required about acupressure treatments, such as the use of burn clothing? |  |  |  |  |  |
| **7** | How much information is required to learn how to massage to treat a child's scar? |  |  |  |  |  |
| Taking a bath | | Not at all | Little | To some extent | Rather much | Very much |
| 1 | How much information is required about the importance of bathing in healing burn wounds? |  |  |  |  |  |
| 2 | How much information is required about the frequency of bathing for a burned child? |  |  |  |  |  |
| 3 | How much information is required about how to bathe a child's wound dressing area? |  |  |  |  |  |
| **Getting dressed** | | Not at all | Little | To some extent | Rather much | Very much |
| 1 | How much information is required about the suitable clothes types for the child? |  |  |  |  |  |
| 2 | How much information is required about choosing the right clothes for sun protection? |  |  |  |  |  |
| **physical exercise** | | Not at all | Little | To some extent | Rather much | Very much |
| 1 | How much information is required about the importance of exercising the child after discharge? |  |  |  |  |  |
| 2 | How much information is required about the appropriate exercise types and body movements to reduce limitations and burn scars? |  |  |  |  |  |
| 3 | How much information is required about how to exercise a burned child? |  |  |  |  |  |
| 4 | How much information is required about the procedures required when increasing pain after exercising? |  |  |  |  |  |
| **Nutrition** | | Not at all | Little | To some extent | Rather much | Very much |
| 1 | How much information is required about the importance of nutrition in wound healing? |  |  |  |  |  |
| 2 | How much information is required about food groups helpful in healing burn wounds? |  |  |  |  |  |
| 3 | How much information is required to treat the child's anorexia? |  |  |  |  |  |
| 4 | How much information is required about the child's daily diet? |  |  |  |  |  |
| 5 | How much information is required about harmful or forbidden foods? (Considering the child's underlying diseases, such as diabetes and fauvism). |  |  |  |  |  |
| **Itch** | | Not at all | Little | To some extent | Rather much | Very much |
| 1 | How much information is required about ways to reduce itching? |  |  |  |  |  |
| **Pain** | | Not at all | Little | To some extent | Rather much | Very much |
| 1 | How much information is required about ways to reduce the pain? |  |  |  |  |  |
| 2 | How much information is required about the required actions to take when increasing pain following exercise? |  |  |  |  |  |
| **Follow-up** | | Not at all | Little | To some extent | Rather much | Very much |
| 1 | How much information is required about participating in post-discharge rehabilitation programs? |  |  |  |  |  |
| 2 | How much information is required about making an appointment at a burn clinic? |  |  |  |  |  |
| **Psychosocial disorders** | | Not at all | Little | To some extent | Rather much | Very much |
| 1 | How much information is required about ways to treat the child's distorted body images? |  |  |  |  |  |
| 2 | How much psychiatric counseling is required? |  |  |  |  |  |
| 3 | How much information is required about reducing the stress and anxiety caused by the burn consequences? |  |  |  |  |  |
| 4 | How much information is required about improving the child's communication skills? |  |  |  |  |  |
| 5 | How much information is required to improve the child's quality of life? |  |  |  |  |  |
| 6 | How much information is required about the child's rehabilitation and recovery status? |  |  |  |  |  |
| 7 | How much information is required about improving the child's sleep quality? |  |  |  |  |  |
| 8 | How much information is required about how to access the burn support organizations? |  |  |  |  |  |
