# Supplementary material for: Health care needs, eHealth literacy, use of mobile phone functionalities, and intention to use it for self-management purposes by informal caregivers of children with burns: a survey study
Source: BMC Med Inform Decis Mak. 2023 Oct 23;23:236. doi: 10.1186/s12911-023-02334-w (PMC10591411; doi:10.1186/s12911-023-02334-w)
Supplement: Supplementary file 2 — Additional file 2: Use of mobile phone functionalities and intention to use for self-management purposes by caregivers of children with burns. [file 12911_2023_2334_MOESM2_ESM.docx]

**Additional file 2: Use of mobile phone functionalities and intention to use for self-management purposes by caregivers of children with burns**

**Demographic characteristics**

**Year of Birth (Caregiver):** ………………. **Year of Birth (children):** ……………….

**Marital status:**  Single  Married  other

**Gender**:  Female  Male

**Place of residence:**  City  Village

**The economic status of the family:**  Low  Moderate  Excellent

**Level of education:**  High School or lower  Diploma's degree  Bachelor’s degree

Associate's degree  Master's degree or higher

**Burn percent (TBSA):** ……………….  **Cause of burn:** ……………….

1. Do you use a mobile phone to receive burn care services?  Yes  No
2. How often do you use mobile phone calls (to friends, relatives, doctors, and nurses) to receive burn-related information?

Everyday  Several Times per Week  Occasionally  Never

1. How often do you use SMS (to friends, relatives, doctors and nurses) to receive burn-related information?

Everyday  Several Times per Week  Occasionally  Never

1. Do you have internet access through your mobile phone?  Yes  No
2. How often do you use your mobile internet to search for burn-related information?

Everyday  Several Times per Week  Occasionally  Never

1. How often do you use social media (such as Telegram channels) to access burn-related information?

Everyday  Several Times per Week  Occasionally  Never

1. How often do you use mobile email to communicate with others (friends, relatives, doctors and nurses) to receive burn-related information?

Everyday  Several Times per Week  Occasionally  Never

1. Do you have a smartphone (iPhone/Android etc.)?  Yes  No
2. Do you have burn-related apps on your mobile phone?  Yes  No
3. How often do you use mobile apps (software) to access burn-related information?

Everyday  Several Times per Week  Occasionally  Never

* Which mobile phone functionality do you **currently use** to receive burn care services (from a doctor or nurse)? (You can also select several options). Other items please be mentioned here. …………………...

| Burn care services | | Mobile phone functionalities | | | | | | | |
| --- | --- | --- | --- | --- | --- | --- | --- | --- | --- |
|  |  | None | Phone/Voice Call | SMS | Internet Search | Social Media | Email | Software/Apps | Video Call |
| 11 | Receiving information about **dressings** |  |  |  |  |  |  |  |  |
| 12 | Receiving information about control of infection and wound care |  |  |  |  |  |  |  |  |
| 13 | Receiving information about taking a bath of wound area and scar |  |  |  |  |  |  |  |  |
| 14 | Receiving information about the importance of the clothes the child wears |  |  |  |  |  |  |  |  |
| 15 | Receiving information about physical exercise |  |  |  |  |  |  |  |  |
| 16 | Receiving information about feeding |  |  |  |  |  |  |  |  |
| 17 | Receiving information about itch |  |  |  |  |  |  |  |  |
| 18 | Receiving information about Pain |  |  |  |  |  |  |  |  |
| 19 | Receiving information about Psychosocial disorders |  |  |  |  |  |  |  |  |
| 20 | Receiving information about drug |  |  |  |  |  |  |  |  |
| 21 | Communicating with other caregivers |  |  |  |  |  |  |  |  |
| 22 | Reminders about doctor or nurse appointments |  |  |  |  |  |  |  |  |
| 23 | Reminders for medication use |  |  |  |  |  |  |  |  |
| 24 | Warning about lack of rehabilitation program |  |  |  |  |  |  |  |  |

25. Would you like to use the mobile phone to receive burn care services? Yes  No the reason for your unwillingness.........................

* Which mobile phone functionality would you like to receive burn care services (from a doctor or nurse)? (You can also select several options). Other items please be mentioned here. …………………...

| Burn care services | | Mobile phone functionalities | | | | | | | |
| --- | --- | --- | --- | --- | --- | --- | --- | --- | --- |
|  |  | None | Phone/Voice Call | SMS | Internet Search | Social Media | Email | Software/Apps | Video Call |
| 26 | Receiving information about **dressings** |  |  |  |  |  |  |  |  |
| 27 | Receiving information about control of infection and wound care |  |  |  |  |  |  |  |  |
| 28 | Receiving information about taking a bath of wound area and scar |  |  |  |  |  |  |  |  |
| 29 | Receiving information about the importance of what the child wears (clothes) |  |  |  |  |  |  |  |  |
| 30 | Receiving information about physical exercise |  |  |  |  |  |  |  |  |
| 31 | Receiving information about feeding |  |  |  |  |  |  |  |  |
| 32 | Receiving information about itch |  |  |  |  |  |  |  |  |
| 33 | Receiving information about Pain |  |  |  |  |  |  |  |  |
| 34 | Receiving information about Psychosocial disorders |  |  |  |  |  |  |  |  |
| 35 | Receiving information about drug |  |  |  |  |  |  |  |  |
| 36 | Communicating with other caregivers |  |  |  |  |  |  |  |  |
| 37 | Reminders about doctor or nurse appointments |  |  |  |  |  |  |  |  |
| 38 | Reminders for medication use |  |  |  |  |  |  |  |  |
| 39 | Warning about lack of rehabilitation program |  |  |  |  |  |  |  |  |
