# Supplementary material for: Health care needs, eHealth literacy, use of mobile phone functionalities, and intention to use it for self-management purposes by informal caregivers of children with burns: a survey study
Source: BMC Med Inform Decis Mak. 2023 Oct 23;23:236. doi: 10.1186/s12911-023-02334-w (PMC10591411; doi:10.1186/s12911-023-02334-w)
Supplement: Supplementary file 3 — Additional file 3: eHealth Literacy Questionnaire. [file 12911_2023_2334_MOESM3_ESM.docx]

**Additional file 3: eHealth Literacy Questionnaire**

| **Strongly Agree** | **Agree** | **Undecided** | **Disagree** | **Strongly Disagree** | **Items** | |
| --- | --- | --- | --- | --- | --- | --- |
|  |  |  |  |  | I know **what** health resources are available on the Internet | 1 |
|  |  |  |  |  | I know **where** to find helpful health resources on the Internet | 2 |
|  |  |  |  |  | I know **how** to find helpful health resources on the Internet | 3 |
|  |  |  |  |  | I know **how to use** the Internet to answer my questions about health | 4 |
|  |  |  |  |  | I know how to use **the health information** I find on the Internet to help me | 5 |
|  |  |  |  |  | I have the skills I need to **evaluate** the health resources I find on the Internet | 6 |
|  |  |  |  |  | I can tell **high high-quality** resources from **low low-quality** resources on the Internet | 7 |
|  |  |  |  |  | I feel **confident** in using information from the Internet to make health decisions | 8 |
