# Supplementary material for: Health care needs, eHealth literacy, use of mobile phone functionalities, and intention to use it for self-management purposes by informal caregivers of children with burns: a survey study
Source: BMC Med Inform Decis Mak. 2023 Oct 23;23:236. doi: 10.1186/s12911-023-02334-w (PMC10591411; doi:10.1186/s12911-023-02334-w)
Supplement: Supplementary file 4 — Additional file 4: Use and desire to use mobile phone functionalities for receiving information, as reminders and warnings in caregivers of children with burns (N=112). [file 12911_2023_2334_MOESM4_ESM.doc]

**Additional file 4:** Use and desire to use mobile phone functionalities for receiving information, as reminders and warnings in caregivers of children with burns (N=112)

| Use of mobile phone functionalities | | | | | | | |
| --- | --- | --- | --- | --- | --- | --- | --- |
| **Items** | **None** | **Phone/ Voice Call** | **SMS** | **Email** | **Internet Search** | **Social Media** | **Software/Applications** |
| 1. Receiving information about **dressings** | 68 (60.7) | 19 (17.0) | 8 (7.1) | 0 | 27 (24.1) | 15 (13.4) | 0 |
| 2. Receiving information about control of infection and wound care | 41 (36.6) | 33 (29.5) | 10 (8.9) | 0 | 59 (52.7) | 46 (41.1) | 0 |
| 3. Receiving information about taking baths of wound area and scar | 67 (59.8) | 21 (18.8) | 6 (5.4) | 1 (0.9) | 38 (33.9) | 19 (17.0) | 0 |
| 4. Receiving information about the importance of the clothes the child wears | 57 (50.9) | 18 )16.1) | 6 (5.4) | 0 | 39 (34.8) | 23 (20.5) | 0 |
| 5. Receiving information about physical exercise | 56 (50.0) | 9 (8.0) | 3 (2.7) | 0 | 49 (43.8) | 38 (33.9) | 0 |
| 6. Receiving information about feeding | 59 (52.7) | 22 (19.6) | 10 (8.9) | 0 | 45 (40.2) | 32 (28.6) | 0 |
| 7. Receiving information about itch | 42 (37.5) | 47 (42.0) | 14 (12.5) | 0 | 50 (44.6) | 35 (31.3) |  |
| 8. Receiving information about Pain | 36 (32.1) | 49 (43.8) | 18 (16.1) | 0 | 57 (50.9) | 41 (36.6) | 0 |
| 9. Receiving information about Psychosocial disorders | 33 (29.5) | 53 (47.3) | 23 (20.5) | 0 | 60 (53.6) | 34 (30.4) | 0 |
| 10. Receiving information about drug | 73 (65.2) | 20 (17.9) | 5 (4.5) | 0 | 23 (20.5) | 6 (5.4) | 0 |
| 11. Communicating with other caregivers | 77 (68.8) | 15 (13.4) | 6 (5.4) | 0 | * | 0 | 1 (0.9) |
| 12. Reminders about doctor or nurse appointments | 70 (62.5) | 21 (18.8) | 6 (5.4) | 0 | * | 27 (24.1) | 0 |
| 13. Medication use reminder | 93 (83.0) | 9 (8.3) | 4 (3.6) | 0 | * | 14 (12.5) | 0 |
| 14. Warning about lack of rehabilitation program | 91 (81.3) | 6 (5.4) | 9 (8.0) | 0 | * | 7 (6.3) | 0 |
| **Desire** t**o u**se mobile phone functionalities | | | | | | | |
| **Items** | **None** | **Phone/Voice Call** | **SMS** | **Email** | **Internet Search** | **Social Media** | **Software/Applications** |
| 1. Receiving information about kind of **dressings** | 29 (26.9) | 27 (24.1) | 10 (8.9) | 0 | 42 (37.5) | 33 (29.5) | 45 (40.2) |
| 2. Receiving information about control of infection and wound care | 23 (20.5) | 40 (35.7) | 15 (13.4) | 0 | 65 (58.0) | 56 (50.0) | 52 (46.4) |
| 3. Receiving information about taking baths of wound area and scar | 37 (33.0) | 27 (24.1) | 8 (7.1) | 1 (0.9) | 50 (44.6) | 37 (33.0) | 45 (40.2) |
| 4. Receiving information about the importance of the clothes the child wears | 52 (46.4) | 20 (17.9) | 5 (4.5) | 0 | 40 (35.7) | 33 (29.5) | 39 (34.8) |
| 5. Receiving information about physical exercise | 20 (17.9) | 22 (19.6) | 7 (6.3) | 0 | 62 (55.4) | 55 (49.1) | 59 (52.7) |
| 6. Receiving information about feeding | 37 (33.0) | 18 (16.1) | 5 (4.5) | 0 | 61 (54.5) | 51 (45.5) | 48 (42.9) |
| 7. Itching | 40 (35.7) | 35 (31.3) | 11 (9.8) | 0 | 60 (53.6) | 40 (35.7) | 47 (42.0) |
| 8. Pain | 38 (33.9) | 36 (32.1) | 9 (8.0) | 0 | 60 (53.6) | 44 (39.3) | 47 (42.0) |
| 9. Receiving information about psychologic disorder | 32 (28.6) | 54 (48.2) | 16 (14.3) | 0 | 57 (50.9) | 50 (44.6) | 48 (42.9) |
| 10. Receiving information about drug | 37 (33.0) | 18 (16.1) | 16 (14.3) | 1 (0.9) | 45 (40.2) | 15 (13.4) | 41 (36.6) |
| 11. Communicating with other caregivers | 45 (40.2) | 35 (31.3) | 24 (21.4) | 0 | * | 34 (30.4) | 27 (25.0) |
| 12. Reminders about doctor or nurse appointments | 33 (29.5) | 24 (21.4) | 57 (50.9) | 0 | * | 31 (27.7) | 35 (31.3) |
| 13. Medication use reminder | 32 (28.6) | 18 (16.1) | 55 (49.1) | 0 | * | 34 (30.4) | 45 (40.2) |
| 14. Warning about not doing a rehabilitation program | 36 (32.1) | 17 (15.2) | 54 (48.2) | 0 | * | 38 (33.9) | 50 (44.6) |

Data are presented as number (percentage).
