# Supplementary material for: Health care needs, eHealth literacy, use of mobile phone functionalities, and intention to use it for self-management purposes by informal caregivers of children with burns: a survey study
Source: BMC Med Inform Decis Mak. 2023 Oct 23;23:236. doi: 10.1186/s12911-023-02334-w (PMC10591411; doi:10.1186/s12911-023-02334-w)
Supplement: Supplementary file 5 — Additional file 5: Distribution and frequency of eHealth Literacy Scale (eHEALS) scores. [file 12911_2023_2334_MOESM5_ESM.docx]

**Additional file 5: Distribution and frequency of eHealth Literacy Scale (eHEALS) scores**

| Response | | | eHEALS items | |
| --- | --- | --- | --- | --- |
| 4-5 | 3 | 1-2 |  |  |
| 71 (63.4) | 7 (6.3) | 34 (30.4( | I know what health resources are available on the Internet | 1 |
| 75 (67.0) | 4 (3.6) | 33 (29.5( | I know where to find helpful health resources on the Internet | 2 |
| 73 (65.2) | 5 (4.5) | 34 (30.4) | I know how to find helpful health resources on the Internet | 3 |
| 68 (60.7) | 8 (7.1) | 36 (32.1) | I know how to use the Internet to answer my questions about health | 4 |
| 65 (58.0) | 7 (6.3) | 40 (35.7) | I know how to use the health information I find on the Internet to help me | 5 |
| 46 (41.1) | 7 (6.3) | 59 (52.7) | I have the skills I need to evaluate the health resources I find on the Internet | 6 |
| 41 (36.6) | 10 (8.9) | 61 (54.5) | I can tell high-quality health resources from low-quality health resources on the Internet | 7 |
| 43 (38.4) | 24 (21.4) | 45 (40.2) | I feel confident in using information from the Internet to make health decisions | 8 |
| For items 1–8, response options ranged from 1 “Strongly disagree” to 5 “Strongly agree” | | | | |
